# Effect of Opicapone on Levodopa Pharmacokinetics in Patients with Fluctuating Parkinson's Disease

**DOI:** 10.1002/mds.29193

**Published:** 2022-08-31

**Authors:** Joaquim J. Ferreira, Werner Poewe, Olivier Rascol, Fabrizio Stocchi, Angelo Antonini, Joana Moreira, Bruno Guimarães, José‐Francisco Rocha, Patrício Soares‐da‐Silva

**Affiliations:** ^1^ Laboratory of Clinical Pharmacology and Therapeutics, Faculdade de Medicina Universidade de Lisboa Lisbon Portugal; ^2^ CNS‐Campus Neurológico Sénior Torres Vedras Portugal; ^3^ Department of Neurology Medical University of Innsbruck Innsbruck Austria; ^4^ Department of Neurosciences and Clinical Pharmacology University of Toulouse Toulouse France; ^5^ Department of Neurology University San Raffaele and IRCCS (Istituto di Ricovero e Cura a Carattere Scientifico) San Raffaele Pisana Rome Italy; ^6^ Parkinson and Movement Disorders Unit, Study Center for Neurodegeneration Department of Neurosciences University of Padova Padova Italy; ^7^ BIAL–Portela & C^a^ S.A, Research & Development Department Coronado Portugal; ^8^ University of Porto, Pharmacology Department Porto Portugal; ^9^ MedInUP–Center for Drug Discovery and Innovative Medicines University of Porto Porto Portugal

**Keywords:** opicapone, levodopa, pharmacokinetics, motor response, COMT inhibitors

## Abstract

**Background:**

Inhibiting catechol‐O‐methyltransferase extends the plasma half‐life of levodopa, potentially allowing physicians to optimize the levodopa regimen in patients with Parkinson's disease (PD) experiencing motor fluctuations.

**Objectives:**

To evaluate the effects of once‐daily opicapone on levodopa plasma pharmacokinetics and motor response when added to two different levodopa dosing regimens.

**Methods:**

A total of 24 patients with PD and motor fluctuations were enrolled in an exploratory, open‐label, modified cross‐over trial. Participants first received levodopa/carbidopa 500/125 mg (five intakes) for 2 weeks and were then randomly assigned (1:1) to levodopa/carbidopa 400/100 mg given over either four or five daily intakes plus opicapone 50 mg for an additional 2 weeks. Levodopa 12‐hour pharmacokinetics was the primary outcome (ie, excluding the effect of last/evening levodopa/carbidopa intake), with motor complications evaluated as secondary outcomes.

**Results:**

Over 12‐hour pharmacokinetics and compared with five‐intake levodopa/carbidopa 500/125 mg without opicapone, maximal levodopa concentrations were similar or nonsignificantly higher on both levodopa/carbidopa 400/100 mg regimens plus opicapone. Despite a 100 mg lower total levodopa/carbidopa daily dose, adding opicapone 50 mg at least doubled the levodopa plasma half‐life and minimal concentrations, with a significant ≈30% increase in total exposure. The levodopa fluctuation index was only significantly lower for the five intakes plus opicapone regimen (difference of −71.8%; *P* < 0.0001). Modifications to levodopa pharmacokinetics were associated with decreased *off* time and increased *on* time.

**Conclusions:**

Combining opicapone 50 mg with a 100 mg lower daily dose of levodopa provides higher levodopa bioavailability with avoidance of trough levels. Despite the lower levodopa dose, modifying the levodopa pharmacokinetic profile with opicapone was associated with decreased *off* time and increased *on* time. © 2022 The Authors. *Movement Disorders* published by Wiley Periodicals LLC on behalf of International Parkinson and Movement Disorder Society

## Graphical abstract

Adjustments of levodopa dose size and/or frequency are common approaches to manage response fluctuations. However, the addition of opicapone in lower levodopa doses resulted in significant increases in levodopa exposure, leading to improved clinical outcomes. This will allow physicians to individually adjust the levodopa regimen that best suits patients' needs.

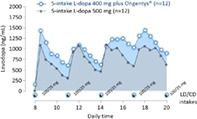

There is agreement that levodopa (LD) is the gold standard treatment for Parkinson's disease (PD) and that virtually all patients will require its unrivaled symptomatic efficacy during their disease management.^1^, ^2^, ^3^ Although LD is well tolerated and efficacious, its clinical utility over time is often limited by the development of response fluctuations, such as wearing‐off and motor fluctuations, and other LD‐induced complications, such as dyskinesia.^4^ The short half‐life of LD (≈90 minutes) and its erratic gastrointestinal absorption are key factors responsible for the fluctuating plasma concentrations of LD,^5^, ^6^, ^7^, ^8^ which give rise to pre‐ and postsynaptic changes in dopaminergic function in the striatum and decreased control of dopaminergic transmission at the synaptic level.^5^, ^6^, ^9^, ^10^, ^11^, ^12^

Adjustments of LD dose size and/or frequency are common approaches to manage response fluctuations.^13^ However, increasing the LD total daily dose by increasing dosing frequency may worsen the severity of dyskinesia, whereas fragmenting the total daily dose into more frequent, smaller doses may be associated with the intermittent reemergence of symptoms attributed to oscillations of plasma concentration over and below the threshold governing LD clinical response.^14^ As neither approach addresses the issue of the short half‐life of conventional LD in the long‐term, an alternative pharmacological approach is to optimize LD delivery to the brain and manage LD‐related complications by administering LD/dopa decarboxylase inhibitor (DDCi) with catechol‐O‐methyltransferase (COMT) inhibitors that increase the plasma half‐life of LD, thus extending the duration of clinical effect.^15^, ^16^ COMT inhibition is key to determining the amount of LD reaching the brain and the extent and duration of its therapeutic effect.

Opicapone, a third‐generation, once‐daily COMT inhibitor, was developed to fulfill the need for a more potent, longer acting COMT inhibitor.^5^, ^6^ In the opicapone pivotal trials, opicapone reduced *off* time by an average of 1 hour relative to placebo; a significant increase in *on* time without dyskinesias was also observed without a significant increase in troublesome dyskinesias.^17^, ^18^ In patients with PD, the administration of opicapone was shown to increase LD bioavailability and trough concentrations with decreased peak‐to‐trough fluctuations.^9^, ^16^, ^19^

In prior clinical studies,^17^, ^18^ patients entered on a wide variety of LD regimens, and the effect of the LD treatment regimen (both total daily dose and frequency of daily intakes) on the clinical effectiveness of opicapone in the management of motor fluctuations is still unknown. Understanding the role of LD total and individual doses and the frequency of intakes is important for optimizing pharmacological adjunctive treatments in the management of motor fluctuations. This study aimed to evaluate the effects of opicapone on LD pharmacokinetics and motor fluctuations in two fixed and arbitrarily defined 400 mg LD plus opicapone 50 mg treatment regimens when compared with 500 mg LD (distributed in five daily intakes each of 100 mg) without opicapone.

## Methods

This was a phase 2, randomized, open‐label study of once‐daily opicapone as adjunct to different LD/carbidopa (CD) dose regimens in patients with PD and end‐of‐dose motor fluctuations conducted between January 12, 2021, and July 13, 2021 (EudraCT number: 2020–003139‐12).

### Study Population

Eligible patients were men and women aged ≥30 years with a clinical diagnosis of idiopathic PD^20^ and disease severity stages I to III (modified Hoehn & Yahr staging) at *on* and signs of “wearing‐off.” Patients had to be treated with LD/DDCi for at least 1 year with clear clinical improvement. Although there were no specific requirements in terms of LD dose frequency, all patients recruited were already receiving a daily treatment regimen of five‐intake LD/CD 500/125 mg. Patients were excluded if they were experiencing severe and/or unpredictable *off* periods, had received treatment with sustained release LD/DDCI in the past 4 weeks prior to screening, were taking prohibited medication (neuroleptics, venlafaxine, monoamine oxidase [MAO] inhibitors [except selegiline, safinamide, or rasagiline], or antiemetics with antidopaminergic action [except domperidone]), or if they had previously received any COMT inhibitor (entacapone, tolcapone, or opicapone).

### Study Design

**FIG 1:**
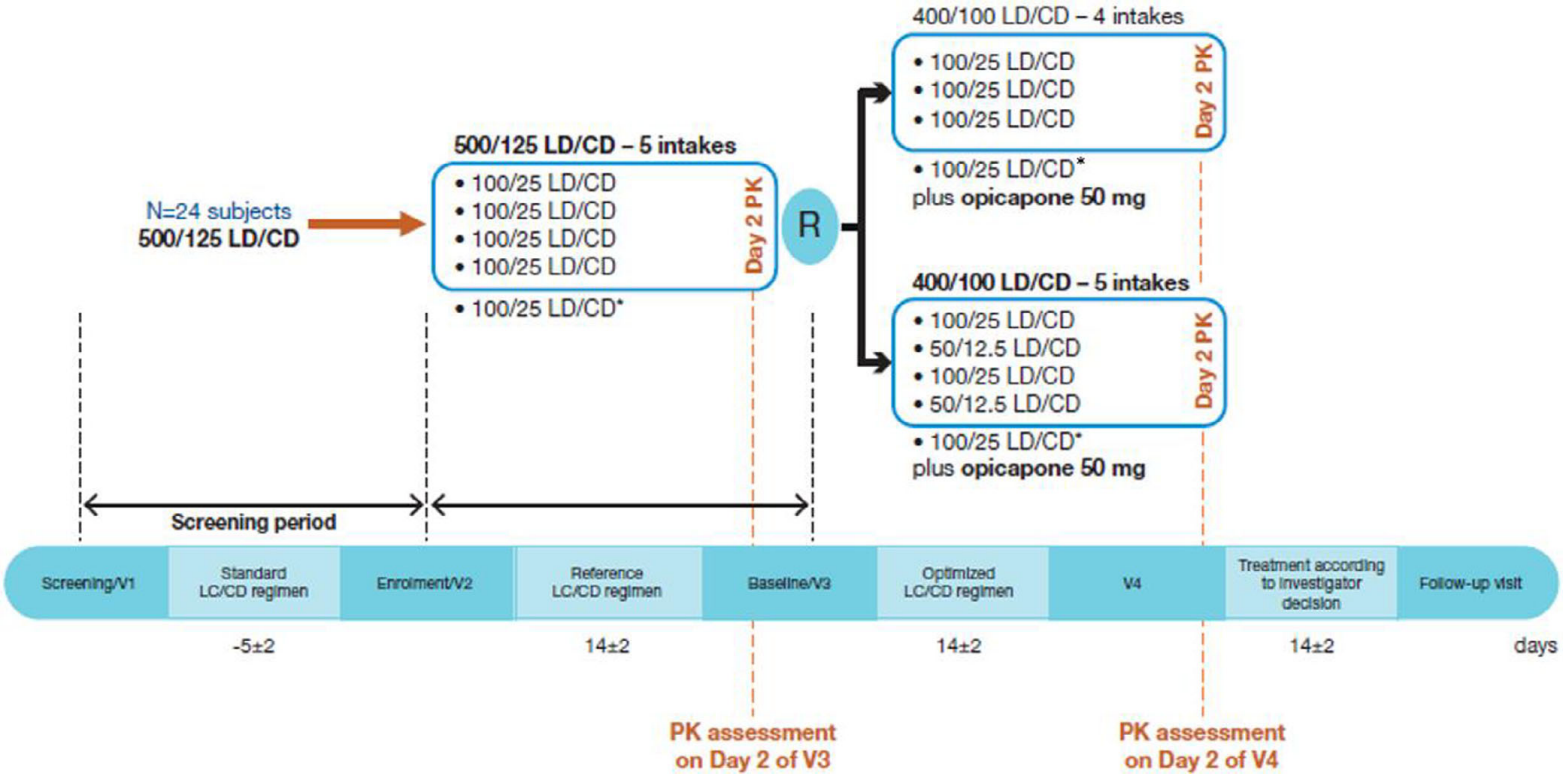
Study design. *Not included in the pharmacokinetics assessment. CD, carbidopa; LD, levodopa; PK, pharmacokinetics; R, randomization; V, visit. [Color figure can be viewed at wileyonlinelibrary.com]

The study design is illustrated in Figure 1. Following screening, all patients received a daily LD/CD dose of 500/125 mg for 2 weeks, administered as LD/CD 100/25 mg five times a day (five‐intake LD/CD 500/125 mg), fixed every 3 hours (from 8 am to 8 pm) at pharmacokinetic day. Patients were then randomly assigned (1:1) to receive two different regimens of opicapone 50 mg plus LD/CD (400/100 mg daily) for an additional 2 weeks.

- Patients on opicapone regimen 1 received LD/CD 100/25 mg four times a day plus opicapone 50 mg at least 1 hour after the last administration of LD/CD each day (four‐intake LD/CD 400/100 mg plus opicapone 50 mg), fixed every 4 hours (from 8 AM to 8 PM) at pharmacokinetic day.
- Patients on opicapone regimen 2 received alternating LD/CD 100/25 mg and 50/12.5 mg doses (ie, 100/25 mg, 50/12.5 mg, 100/25 mg, 50/12.5 mg, and 100/25 mg) five times a day plus opicapone 50 mg at least 1 hour after the last administration of LD/CD each day (five‐intake LD/CD 400/100 mg plus opicapone 50 mg), fixed every 3 hours (from 8 AM to 8 PM) at pharmacokinetic day.

After completing the two 2‐week LD/CD 400/100 mg plus opicapone 50 mg regimens, patients were followed up for 1 to 2 weeks (poststudy visit). The total duration of study participation was 7 to 8 weeks.

### Study Assessments

The primary objective of the study was to evaluate the pharmacokinetics of LD in the two opicapone LD/CD 400/100 mg dosing regimens (four and five intakes) when compared with the five‐intake LD/CD 500/125 mg regimen without opicapone. The pharmacokinetics study endpoint was set after 2 weeks of each treatment regimen (ie, at the end of the initial five‐intake LD/CD 500/125 mg treatment regimen and at the end of the LD/CD 400/100 mg plus opicapone treatment). Pharmacokinetic evaluation occurred over 12 hours with sampling every 30 minutes, except for the last sample following each LD dose in the opicapone plus four LD intake regimen, which was sampled at 1 hour. The main pharmacokinetics parameters assessed included maximum observed plasma concentration (C_max_), time to achieve peak plasma levels (t_max_), minimum observed plasma concentration (C_min_), plasma elimination half‐life (t_1/2_), area under the curve (AUC), and fluctuation index (FI; as an expression of peak‐to‐trough fluctuation, C_max_, and C_min_, relative to average concentration [C_avg_]). The pharmacokinetics of the LD metabolite 3‐O‐methyldopa (3‐OMD) was also evaluated.

Clinical and safety/tolerability outcomes were also assessed as exploratory secondary outcomes. Clinical outcomes included *on* and *off* time assessed using patient‐rated tools. The timing of *on* and *off* states was registered (in real time) by the investigators during the matching 12‐hour pharmacokinetic evaluation (as such applied on pharmacokinetics days only), and patients also completed 24‐hour patient Hauser *on*/*off* diary charts^21^ (in 30‐minute blocks) during the 3 days before each pharmacokinetic visit. Diary states of interest were *off* time and *on* time, including *on* without dyskinesia, *on* with nontroublesome dyskinesia, and *on* with troublesome dyskinesia. In addition, the Patient Global Impression of Change (PGI‐C) of opicapone 50 mg was evaluated at the end of the LD/CD 400/100 mg plus opicapone treatment compared with before the start of opicapone treatment.^22^ Safety and tolerability endpoints included treatment‐emergent adverse events (TEAEs), serious TEAEs, and TEAEs leading to discontinuation.

All study assessments were evaluated for each LD/CD 400/100 mg plus opicapone treatment regimen compared with the LD/CD 500/125 mg without opicapone regimen.

### Ethical Approval and Consent

The clinical study protocol and the informed consent form were reviewed and approved by the independent ethics committees of the participating sites. All patients included in the study signed a patient consent form.

### Statistical Analyses

#### Pharmacokinetics Parameters

As this was an exploratory study in nature, no formal sample size calculation was performed, but 12 patients per treatment group were considered sufficient for a pharmacokinetics study.

Descriptive statistics were used to summarize the pharmacokinetics parameters for LD and 3‐OMD for each of the three regimens. LD and 3‐OMD AUC_total_ (AUC to the last measurable time point of the last LD/CD dose), C_max,max_ (maximum C_max_ observed during the first three or four LD/CD doses), C_min,min_ (minimum C_min_ observed during the first three or four LD/CD doses, excluding first predose data), and FI were also summarized for each treatment arm based on all patients with estimatable data (patients must have had a minimum of three data points after C_max_ to estimate the terminal elimination rate constant required to estimate the t_1/2_, C_avg_, area under the concentration‐time curve over the dosing interval, and FI percentage). No formal comparison was performed between opicapone regimens. Pharmacokinetic parameters were evaluated within the same population for each LD/CD 400/100 mg plus opicapone treatment regimen compared with the LD/CD 500/125 mg without opicapone regimen. Geometric mean ratios and corresponding 90% confidence intervals (CIs) for the LD and 3‐OMD log‐transformed pharmacokinetics parameters (AUC_total_, C_max,max_ and C_min,min_ [only for LD]) were calculated using a paired *t* test for the comparison between the five‐intake LD/CD 500/125 mg treatment without opicapone and each of the two LD/CD 400/100 mg plus opicapone regimens; only data from completers (patients with data for both regimens) were used for this analysis.

#### Clinical Outcomes

Clinical outcomes are also presented descriptively. A paired *t* test was used for the comparison of 12‐hour *off* and *on* time, 24‐hour *off* and *on* time, time to on, and time to best *on* between each opicapone‐containing regimen and the regimen without opicapone. Estimates of mean difference and corresponding 90% CIs were reported for all main variables.

## Results

### Study Population

In total, 25 patients were recruited, but one did not meet the inclusion criteria and was excluded at screening; the remaining 24 patients completed the study and were included in all analyses. Baseline demographics and disease characteristics were similar between treatment arms (Table 1). In the overall population, the mean age was 62.2 years, the mean duration of PD was 6.6 years, and the mean daily *off* time was 7.3 hours. Approximately 30% of the patients were on LD/CD monotherapy. Overall, 37.5% of patients were taking dopamine agonists, 16.7% were on MAO type B inhibitors, and 4.2% were receiving both dopamine agonists and MAO type B inhibitors as add‐on medications to their LD preparations.

**TABLE 1:** Baseline characteristics

Variable	Five‐intake levodopa/carbidopa 500/125 mg followed by four‐intake levodopa/carbidopa 400/100 mg plus opicapone 50 mg, n = 12	Five‐intake levodopa/carbidopa 500/125 mg followed by five‐intake levodopa/carbidopa 400/100 mg plus opicapone 50 mg, n = 12	Overall, N = 24
Male sex, n (%)	4.0 (33.3)	9.0 (75.0)	13.0 (54.2)
Mean age, y (SD)	63.3 (4.1)	61.0 (9.2)	62.2 (7.1)
Mean weight, kg (SD)	77.6 (13.2)	86.1 (17.9)	81.9 (16.0)
Mean height, cm (SD)	164.7 (11.2)	170.6 (6.6)	167.3 (9.5)
Mean PD duration, y (SD)	7.2 (3.8)	6.0 (2.3)	6.6 (3.2)
Mean daily *off* time, h (SD)	7.4 (1.6)	7.2 (1.7)	7.3 (1.6)
Presence of dyskinesia, n (%)^a^	7 (58.3)	9 (75.0)	16 (66.7)
PD medications given in addition to levodopa/carbidopa, n (%)	9 (75.0)	8 (66.7)	17 (70.8)
Pramipexole	3 (33.3)	3 (37.5)	6 (35.3)
Selegiline	2 (22.2)	3 (37.5)	5 (29.4)
Ropinirole	3 (33.3)	1 (12.5)	4 (23.5)
Trihexyphenidyl	2 (22.2)	1 (12.5)	3 (17.6)
Amantadine	1 (11.1)	2 (25.0)	3 (17.6)
Patients receiving DA and MAO‐Bi^b^ in addition to levodopa/carbidopa, n (%)			
Levodopa/carbidopa plus DA only	6 (50.0)	3 (25.0)	9 (37.5)
Levodopa/carbidopa plus MAO‐Bi only	2 (16.7)	2 (16.7)	4 (16.7)
Levodopa/carbidopa plus DA and MAO‐Bi	0 (0.0)	1 (8.3)	1 (4.2)

^a^ Presence of dyskinesia as assessed by Hauser diary data at baseline.

^b^ Some patients were receiving additional PD drugs that were not DA or MAO‐Bi.

SD, standard deviation; PD, Parkinson's disease; DA, dopamine agonists; MAO‐Bi, monoamine oxidase inhibitors.

### Pharmacokinetics

**FIG 2:**
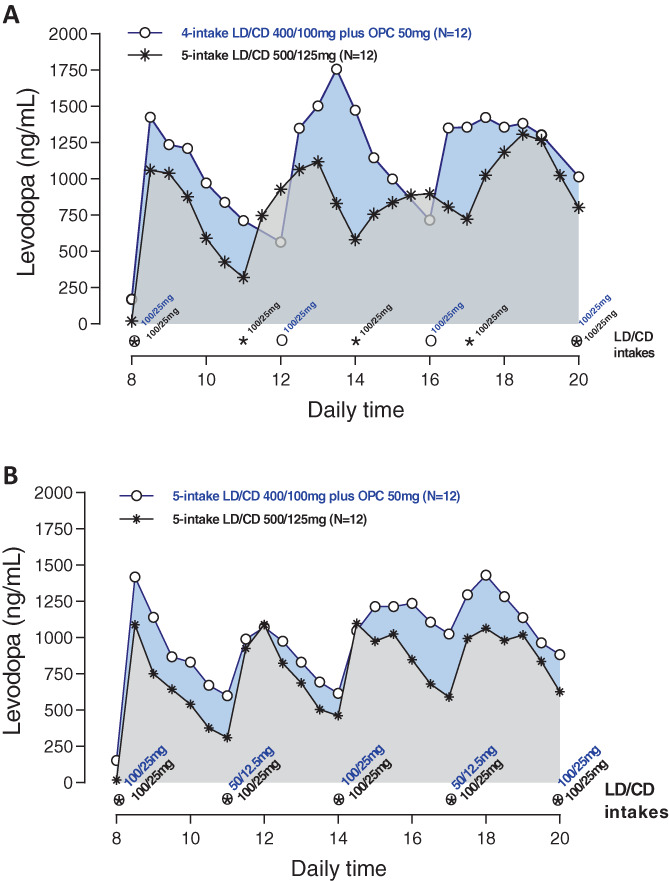
Mean levodopa plasma profile versus time following 2‐week, five‐intake (every 3 hours) daily oral administrations of LD/CD 500/125 mg compared with 2‐week, four‐intake (every 4 hours) daily oral administrations of LD/CD 400/100 mg plus once‐daily opicapone 50 mg (A) or compared with 2‐week, five‐intake (every 3 hours) daily oral administrations of LD/CD 400/100 mg plus once‐daily opicapone 50 mg (B). LD/CD, levodopa/carbidopa; OPC, opicapone. [Color figure can be viewed at wileyonlinelibrary.com]

The 12‐hour pharmacokinetics of LD and 3‐OMD for the two different LD/CD 400/100 mg plus opicapone regimens compared with the five‐intake LD/CD 500/125 mg without opicapone regimen are shown in Figure 2 and Table 2 for LD and in Supporting Information Figure S2 and Table S3 for 3‐OMD.

**TABLE 2:** Levodopa pharmacokinetic parameters following 2‐week, five‐intake (every 3 hours) daily oral administrations of LD/CD 500/125 mg without opicapone compared with 2‐week, four‐intake (every 4 hours) daily oral administrations of LD/CD 400/100 mg plus once‐daily opicapone 50 mg or compared with 2‐week, five‐intake (every 3 hours) daily oral administrations of LD/CD 400/100 mg plus once‐daily opicapone 50 mg

Variable	C_max_ (ng/mL), n = 12	C_max_ (ng/mL), n = 12	t_max_ (h), n = 12	C_min_ (ng/mL), n = 12	C_min_ (ng/mL), n = 12	C_avg_ (ng/mL), n = 12	AUCτ (h · ng/mL), n = 12	AUC_total_ (h · ng/mL), n = 12	t_1/2_ (h), n = 12	FI (%), n = 12	FI (%), n = 12
Five‐intake LD/CD 500/125 mg vs four‐intake LD/CD 400/100 mg plus opicapone 50 mg											
Five‐intake LD/CD 500/125 mg											
First LD/CD intake	1393	2130 (25.8)	0.75	18.6	264.5 (32.8)	692	2076	10,391 (23.4)	1.1^a^	202	166.9 (20.7)
	(26.5)		(0.5–1.5)	(56.2)		(26.1)	(26.1)		(32.4)	(16.3)	
Second LD/CD intake	1751		1.5	266		879^c^	2637^c^		0.9^c^	176^c^	
	(34.6)		(0.5–2.5)	(31.2)		(28.6)	(28.6)		(27.1)	(34.2)	
Third LD/CD intake	1310		1.5	403		950^f^	2850^f^		1.3^g^	113^f^	
	(36.6)		(0.0–2.9)	(29.5)		(29.3)	(29.3)		(38.4)	(28.3)	
Fourth LD/CD intake	1852		1.5	559		1138^d^	3414^d^		1.2^d^	118^d^	
	(30.5)		(0.5–2.5)	(35.7)		(30.6)	(30.6)		(7.6)	(19.5)	
Four‐intake levodopa/carbidopa 400/100 mg plus opicapone 50 mg											
First LD/CD intake	1814	2511 (34.4)	0.75	221	527.3 (41.1)	926	3705	13,590 (32.8)	2.5	174	147.8 (13.0)
	(33.0)		(0.5–2.0)	(67.9)		(33.1)	(33.1)		(20.3)	(20.9)	
Second LD/CD intake	2235		1.0	533		1212	4849		2.1	144	
	(30.8)		(0.5–2.0)	(38.8)		(31.9)	(31.9)		(18.4)	(23.5)	
Third LD/CD intake	2007		1.75	665		1282^d^	5128^d^		2.7^e^	110^d^	
	(48.5)		(0.5–3.0)	(48.6)		(33.3)	(33.3)		(13.7)	(38.9)	
Five‐intake LD/CD 500/125 mg vs five‐intake LD/CD 400/100 mg plus opicapone 50 mg											
Five‐intake LD/CD 500/125 mg											
First LD/CD intake	1356	1999 (26.3)	0.5	18.1	267.0 (82.9)	596^b^	1788^b^	9310 (23.6)	1.1^c^	231^b^	175.5 (24.9)
	(32.5)		(0.5–2.0)	(37.2)		(20.8)	(20.8)		(27.7)	(17.1)	
Second LD/CD intake	1466		1.0	283		756^b^	2269^b^		1.4^b^	172^b^	
	(32.7)		(0.0–2.9)	(87.3)		(39.6)	(39.6)		(30.9)	(31.7)	
Third LD/CD intake	1530		0.5	365		957^d^	2871^d^		1.5^e^	148^d^	
	(46.0)		(0.0–2.0)	(62.3)		(37.7)	(37.7)		(48.6)	(24.6)	
Fourth LD/CD intake	1540		1.5	455		957^d^	2871^d^		1.4^d^	112^d^	
	(41.6)		(0.5–3.0)	(41.1)		(26.2)	(26.2)		(48.9)	(39.6)	
Five‐intake LD/CD 400/100 mg plus opicapone 50 mg											
First LD/CD intake	1664		0.5	153	578.7 (40.1)	886^a^	2657^a^	12,044 (23.2)	2.1^b^	168^a^	103.7 (16.5)
	(21.8)	1951 (17.7)	(0.5–2.0)	(50.0)		(17.1)	(17.1)		(18.7)	(16.3)	
Second LD/CD intake	1286		1.0	579		799^a^	2397^a^		2.8^a^	88.0^a^	
	(27.1)		(0.5–2.0)	(37.8)		(21.3)	(21.3)		(52.6)	(23.6)	
Third LD/CD intake	1572		1.75	603		1381^f^	4144^f^		2.2^g^	91.6^f^	
	(36.0)		(0.5–2.9)	(35.8)		(22.5)	(22.5)		(28.1)	(28.0)	
Fourth LD/CD intake	1557		1.0	860		1170^b^	3509^b^		2.6^b^	60.2^b^	
	(24.5)		(0.5–2.0)	(25.9)		(17.9)	(17.9)		(36.1)	(27.4)	

All values are expressed as mean (percentage coefficient of variation) except for t_max_ values, which are expressed as median (range). Data are shown for all patients with estimatable parameters: ^a^11 patients, ^b^10 patients, ^c^9 patients, ^d^8 patients, ^e^7 patients, ^f^6 patients, ^g^5 patients.

LD/CD, levodopa/carbidopa; C_max_, maximum observed plasma concentration; t_max_, time taken to reach C_max_; C_min_, minimum observed plasma concentration (excluding first predose); C_avg_, average plasma concentration; AUCτ, area under the concentration‐time curve over the dosing interval; AUC_total_, AUC from zero hours to the last measurable time point; t_1/2_, terminal plasma half‐life; FI, fluctuation index (calculated as: [(C_max_ – C_min_)/C_avg_] × 100).

#### Five‐Intake LD/CD 500/125 mg Without Opicapone Compared with Four‐Intake LD/CD 400/100 mg Plus Opicapone 50 mg

Overall, when compared with the five‐intake LD/CD 500/125 mg without opicapone regimen, the four‐intake LD/CD 400/100 mg plus opicapone 50 mg regimen was characterized by a slightly higher LD C_max_, with a 15% increase in C_max,max_ (maximum C_max_ observed), which was not statistically different; a significantly higher (approximately twofold) LD C_min,min_ (*P* = 0.0016); and a nonsignificant difference in LD t_max_ (of note, following an overnight washout, there was an ≈12‐fold increase in C_min_ before the first LD/CD intake). There was also a twofold higher LD t_1/2_, resulting in a corresponding significant 27% increase in LD AUC_total_ (*P* = 0.0003; Tables 2 and 3). Although the LD FI was reduced versus the five‐intake without opicapone regimen, the reduction of 10% was not statistically significant (difference of −19.1% [90% CI: −37.6, −0.6]; Tables 2 and 3). The four‐intake LD/CD 400/100 mg plus opicapone 50 mg regimen was also associated with a significant 86% decrease in 3‐OMD AUC (*P* < 0.0001; Supporting Information Table S4; Fig. S2A).

**TABLE 3:** Paired *t* test analysis for main levodopa pharmacokinetic and efficacy parameters following 2‐week, five‐intake (every 3 hours) daily oral administrations of levodopa/carbidopa 500/125 mg without opicapone compared with 2‐week, four‐intake (every 4 hours) daily oral administrations of levodopa/carbidopa 400/100 mg plus once‐daily opicapone 50 mg or 2‐week, five‐intake (every 3 hours) daily oral administrations of LD/CD 400/100 mg plus once‐daily opicapone 50 mg

Parameter	N	Geometric mean (90% CI)			Geometric mean ratio		
		Reference (five‐intake levodopa/carbidopa 500/125 mg)	Test (four/five‐intake levodopa/carbidopa 400/100 mg + plus opicapone 50 mg)	Mean difference (90% CI)	Estimate	90% CI	*P* value
Five‐intake levodopa/carbidopa 500/125 mg without opicapone vs four‐intake levodopa/carbidopa 400/100 mg plus opicapone 50 mg							
AUC_total_ (h · ng/mL)	12	10,126 (8940, 11,469)	12,906 (10,810, 15,408)	3199 (1903, 4495)	1.27	1.17, 1.38	0.0003
C_max,max_ (ng/mL)	12	2059 (1782, 2380)	2376 (1980, 2850)	380.8 (−152.3, 914.0)	1.15	0.97, 1.37	0.3053
C_min,min_ (ng/mL)	11	252.2 (211.4, 301.0)	487.1 (387.3, 612.6)	262.7 (133.5, 392.0)	1.93	1.46, 2.55	0.0016
FI (%)	12	163.7 (146.9, 182.3)	146.8 (137.6, 156.6)	−19.11 (−37.62, −0.60)	0.90	0.80, 1.00	0.1095
12‐h time to *on* (min)	12	44.62 (38.75, 51.39)	39.48 (30.41, 51.26)	−1.6 (−13.37, 10.25)	0.88	0.70, 1.12	0.3750
12‐h time to best *on* (min)	12	57.62 (50.84, 65.31)	47.11 (37.79, 58.73)	−8.0 (−16.82, 0.89)	0.82	0.70, 0.96	0.0439
12‐h *off* time (min)	10	356.4 (313.2, 405.5)	299.3 (247.1, 362.5)	−51.5 (−129.4, 26.39)	0.84	0.65, 1.08	0.2367
12‐h *on* time (min)	10	340.3 (292.0, 396.7)	394.3 (347.2, 447.6)	52.5 (−25.84, 130.8)	1.16	0.94, 1.44	0.2394
24‐h total *off* time (min)	12	433.0 (386.4, 485.0)	382.1 (321.0, 454.8)	−42.5 (−69.4, −15.6)	0.88	0.80, 0.97	0.0336
24‐h total *on* time (min)	12	476.5 (421.9, 538.2)	529.3 (473.8, 591.3)	52.5 (29.4, 75.6)	1.11	1.06, 1.16	0.0015
Five‐intake levodopa/carbidopa 500/125 mg without opicapone vs five‐intake levodopa/carbidopa 400/100 mg plus opicapone 50 mg							
AUC_total_ (h · ng/mL)	12	9097 (8111, 10,202)	11,745 (10,386, 13,282)	2734 (2073, 3394)	1.29	1.23, 1.36	<0.0001
C_max,max_ (ng/mL)	12	1931 (1667, 2237)	1920 (1741, 2119)	−48.3 (−248.4, 151.7)	0.99	0.90, 1.10	0.9259
C_min,min_ (ng/mL)	11	218.9 (156.1, 307.0)	542.4 (442.7, 664.5)	311.7 (244.6, 378.8)	2.48	1.90, 3.23	<0.0001
FI (%)	12	169.6 (145.9, 197.1)	102.3 (93.5, 111.9)	−71.8 (−93.4, −50.2)	0.60	0.53, 0.69	<0.0001
12‐h time to *on* (min)	10	41.84 (36.03, 48.59)	27.79 (18.64, 41.44)	−9.3 (−17.84, −0.74)	0.66	0.48, 0.91	0.0420
12‐h time to Best *on* (min)	12	53.97 (47.84, 60.89)	45.99 (37.45, 56.48)	−6.1 (−15.08, −2.91)	0.85	0.72, 1.01	0.1166
12‐h *off* time (min)	9	376.9 (349.9, 406.0)	208.7 (164.2, 265.3)	−157.2 (−203.9, −110.5)	0.55	0.44, 0.70	0.0013
12‐h *on* time (min)	9	333.1 (298.8, 371.2)	489.5 (441.1, 543.1)	158.3 (111.1, 205.6)	1.47	1.32, 1.64	0.0002
24‐h total *off* time (min)	12	420.3 (369.8, 477.6)	318.8 (261.8, 388.3)	−93.3 (−139.6, −47.1)	0.76	0.66, 0.88	0.0056
24‐h total *on* time (min)	12	512.5 (470.9, 557.9)	614.6 (564.2, 669.5)	103.3 (60.97, 145.7)	1.20	1.12, 1.29	0.0007

CI, confidence interval; AUC_total_, area under the curve from zero hours to the last measurable time point; C_max,max_, maximum C_max_ observed; C_min,min_, minimum C_min_ observed (excluding first predose); FI, fluctuation index (calculated as: [(C_max_ – C_min_)/C_avg_] × 100).

#### Five‐Intake LD/CD 500/125 mg Without Opicapone Compared with Five‐Intake LD/CD 400/100 mg Plus Opicapone 50 mg

Overall, when compared with the five‐intake LD/CD 500/125 mg without opicapone regimen, the five‐intake LD/CD 400/100 mg plus opicapone 50 mg regimen was characterized by a significantly higher (≈2.5‐fold) LD C_min,min_ (*P* < 0.0001; of note, following an overnight washout, there was an ≈8.5‐fold increase in C_min_ of the first LD/CD intake) and a more than twofold longer LD t_1/2,_ resulting in a corresponding significant 29% increase in LD AUC_total_ (*P* < 0.0001; Tables 2 and 3). There were no significant differences in C_max_ or t_max_ (Table 3). The stabilized C_max_ together with the significantly increased C_min_ led to a significant 40% lower LD FI ratio (last/evening LD/CD intake was not included in the analysis; *P* < 0.0001; difference of −71.8% [90% CI: −93.4, −50.2]; Table 3). The five‐intake LD/CD 400/100 mg plus opicapone 50 mg regimen was also associated with a significant 86% decrease in 3‐OMD AUC (*P* < 0.0001; Supporting Information Table S4; Fig. S1B).

### Clinical Outcomes

#### Five‐Intake LD/CD 500/125 mg Without Opicapone Compared with Four‐Intake LD/CD 400/100 mg Plus Opicapone 50 mg

##### The 12‐Hour Patient *On/Off* Monitoring

A 16%, nonsignificant decrease in *off* time and a 16% increase in *on* time were observed with four‐intake LD/CD 400/100 mg plus opicapone 50 mg compared with five‐intake LD/CD 500/125 mg without opicapone (Fig. 2, Table 3, and Supporting Information Table S1). Time to *on* and time to best *on* decreased by 12% and 18%, respectively (decrease was significant for time to best *on*; *P* = 0.0439; Table 3).

##### The 24‐Hour Patient Hauser *On/Off* Diary

A significant 12% decrease in total *off* time (*P* = 0.0336; difference of −42.5 minutes [90% CI: −69.4, −15.6]; Table 3) and a significant 11% increase in *on* time (*P* = 0.0015; Table 3) were observed with four‐intake LD/CD 400/100 mg plus opicapone 50 mg compared with five‐intake LD/CD 500/125 mg without opicapone (see additional Supporting Information Table S2). *On* time with troublesome dyskinesia decreased by ≈15% following the four‐intake LD/CD 400/100 mg plus opicapone 50 mg regimen (Supporting Information Table S2).

##### Patient Global Impression of Change

Approximately 70% of patients reported an improvement (very much/much/minimal improvement) on the PGI‐C with the four‐intake LD/CD 400/100 mg plus opicapone 50 mg regimen, with ≈33% of patients experiencing “very much/much improvement” (Supporting Information Fig. S3A).

#### Five‐Intake LD/CD 500/125 mg Without Opicapone Compared with Five‐Intake LD/CD 400/100 mg Plus Opicapone 50 mg

##### The 12‐Hour Patient *On/Off* Monitoring

A significant 45% decrease in *off* time (*P* = 0.0013; Table 3) and a significant 47% increase in *on* time (*P* = 0.0002; Table 3) were observed with five‐intake LD/CD 400/100 mg plus opicapone 50 mg (Fig. 2 and Supporting Information Table S1). Time to *on* and time to best *on* decreased by 34% and 15%, respectively (decrease was significant for time to *on*; *P* = 0.0420; Table 3).

##### The 24‐Hour Patient Hauser *On/Off* Diary

A significant 24% decrease in total *off* time (*P* = 0.0056; difference of −93.3 minutes [90% CI: −139.6, −47.1]; Table 3) and a significant 20% increase in *on* time (*P* = 0.0007; Table 3) were observed with five‐intake LD/CD 400/100 mg plus opicapone 50 mg (see Supporting Information Table S2). *On* time with troublesome dyskinesia decreased by ≈36% (Supporting Information Table S2).

##### Patient Global Impression of Change

Approximately 92% of patients reported an improvement (very much/much/minimal improvement) on the PGI‐C with the five‐intake LD/CD 400/100 mg plus opicapone 50 mg regimen, with 41.7% of patients experiencing “much improvement”; worsening was not reported (Supporting Information Fig. S3B).

### Safety and Tolerability

Two TEAEs (n = 2) were reported for two patients receiving the four‐intake LD/CD 400/100 mg plus opicapone 50 mg regimen (16.7%; Supporting Information Table S5): an increase in blood glucose levels (one event observed in one patient) and an increase in gamma‐glutamyltransferase (GGT) levels (one event observed in one patient). Both events were assessed by the investigator as mild and not related to LD or opicapone and did not result in any changes to the study medication. The patient who reported an increase in blood glucose was diagnosed with type 2 diabetes ≈2 years before study enrollment. The patient who reported an increase in GGT was diagnosed with chronic cholecystitis ≈9 days before study enrollment. At screening, GGT was elevated but not clinically relevant (41 U/L; reference range: 0–38 U/L). At the end of the LD/CD 400/100 mg plus opicapone treatment, GGT was elevated (147 U/L) and recorded as an adverse event (AE); at the poststudy visit, the GGT level was 54 U/L and was considered resolved. Serious TEAEs and discontinuations attributed to TEAEs were not reported for any treatment regimen.

## Discussion

This phase 2 study evaluated the effects of once‐daily opicapone on LD plasma pharmacokinetics and motor response when added to two different LD dosing regimens (400 mg LD, administered in either four or five daily intakes), in comparison with 500 mg LD, administered in five daily intakes. Our results suggest that, despite the lower LD dose (100 mg less/d), adding opicapone 50 mg to LD therapy resulted in a higher LD bioavailability with the avoidance of trough levels when compared with the LD‐only regimen. Despite the limited time period, modifying the pharmacokinetic profile of LD by the addition of opicapone was associated with reduced *off* time and increased *on* time.

In accordance with its known mechanism of action, once‐daily opicapone 50 mg significantly reduced the peripheral metabolism of LD, as also demonstrated by the reduced 3‐OMD plasma levels. Despite a reduced daily LD/CD dose (400/100 mg instead of 500/125 mg LD/CD), opicapone 50 mg at least doubled the t_1/2_ of LD when compared with the LD/CD 500/125 mg without opicapone regimen. The impact on t_1/2_ triggered two immediate consequences: a similar significant twofold increase in LD trough concentrations (C_min_) and a corresponding significant ≈30% increase in LD systemic exposure (AUC_total_). Indeed, our AUC results support the LD equivalent dose conversion factor of 0.5 proposed for opicapone.^23^ Furthermore, adding opicapone 50 mg to either LD/CD 400/100 mg regimen had no impact on the time to reach (t_max_) maximum LD concentrations (C_max_). Although no significant impact on C_max_ was observed when adding opicapone 50 mg to either LD/CD 400/100 mg regimens, a nonsignificantly higher (15%) LD C_max_ was observed with four‐intake LD/CD 400/100 mg plus opicapone 50 mg. A recent cross‐over study of relatively infrequent (4 hour interval) LD dosing found that opicapone increased the LD C_max_,^24^ highlighting the complex interplay between LD dosing and COMT inhibition and lending support to the idea that fractionating into more frequent dosing may be beneficial. The increase in LD C_min_ combined with a steady C_max_ resulted in reduced (up to 40%) LD plasma fluctuations (as assessed by LD fluctuation index), although statistical significance was reached only with five‐intake LD/CD 400/100 mg plus opicapone 50 mg.

The current study was not designed to directly compare the two LD/CD 400/100 mg plus opicapone regimens; however, our results suggest that regimens with the shortest LD dosing interval presented the smoothest LD pharmacokinetic profile. This was observed with the five‐intake LD/CD 400/100 mg plus opicapone 50 mg regimen, as one LD dose of 100 mg was split in two equal intakes administered in a shorter interval. This treatment regimen might allow less fluctuation in the plasma levels of LD when compared with the four‐intake LD/CD regimen with longer dosing intervals. This suggests that more frequent LD daily dosing frequency might offer some advantages in terms of motor fluctuations over less frequent dosing perhaps because of an associated reduction in LD plasma variability. For patients primed for dyskinesia, it is likely that the process of dose regimen optimization will require more fine tuning.

In this study, the effect of opicapone on clinical outcomes was only exploratory; however, it should be noted that the increase in LD trough concentrations was associated with less time needed to achieve the *on* state following each LD dose. Simultaneously, the increase in LD systemic exposure was accompanied by a significant decrease (up to ≈20%) in *off* time (although statistical significance was not achieved for the 12‐hour *off* time monitoring with the four‐intake LD/CD 400/100 mg plus opicapone 50 mg) mirrored in *on* time. Although our interpretations are limited by the short follow‐up duration, the improved pharmacokinetic profile appeared to be associated with a relevant decrease in the *on* time spent by patients with troublesome dyskinesia and also by the lack of reports of dyskinesia as an AE (Fig. S4).

Although ≥4 daily intakes of LD are commonly needed in advanced PD in response to motor fluctuations, there are no formal recommendations on the frequency of LD intakes at the start of LD therapy, which is still usually initiated with three daily doses. Our findings suggest that, in patients with advanced PD, a higher LD dosing frequency is associated with reduced fluctuations of LD levels in the plasma, likely resulting in reduced fluctuations of LD levels in the brain. Similar plasma level oscillations have also been shown to occur in early PD with three daily doses of LD, when they do not yet translate into clinical manifestations. Our findings reemphasize the need for long‐term studies of optimized delivery of LD, including the use of enzyme inhibitors for the prevention or delay of motor complications at the beginning of treatment in LD‐naïve patients.^25^ STRIDE‐PD (STalevo Reduction In Dyskinesia Evaluation) is still the only controlled trial to investigate this possibility and has failed to demonstrate that initiating LD therapy with a COMT inhibitor (entacapone) can delay the time of onset or reduce the frequency of dyskinesia compared with LD‐only therapy.^26^ However, that trial has been criticized for its forced LD dose titration to 400 mg per day and beyond as well as for a fixed four‐times‐daily frequency regimen. It is conceivable that a study design of a similar trial that would adjust for LD equivalent doses and allow more frequent LD dosing might produce a different outcome.

This study has several limitations. First, as it was designed as an open‐label, short‐term, fixed‐sequence, modified cross‐over pharmacokinetic trial, the number of patients was too low to accurately assess clinical outcome. Although the trial design allowed a partial comparison between treatment regimens, a classic cross‐over analysis with a comparison between opicapone regimens was not performed because patients were randomly assigned to receive only one of the two LD plus opicapone 50 mg regimens. Although we did not include a placebo arm, a separate exploratory study is underway to evaluate the effect of adding once‐daily opicapone in patients with PD with early motor fluctuations compared with adding an extra 100 mg dose of LD (standard of care) during a 1‐month evaluation period.^9^ A by‐intake comparison could not be carried out because of the different LD dosing intervals and amount of LD for some daily intakes. Pharmacokinetic sampling was limited to 12 hours because of limits on how much blood could be taken and also because a longer period would require an overnight stay. Thus, we did not check for any accumulation of LD (potentially affecting C_max_) past the 12‐hour assessment period. However, significant increases in C_max_ were observed already after the third and fourth LD doses with entacapone^27^, ^28^ and we did not see this with once‐daily dosing of opicapone in this study. Furthermore, this study was pragmatically designed to include patients who were already receiving treatment with five‐intake LD/CD 500/125 mg. It should be noted that the need to receive this treatment was not designed to mimic the day‐to‐day clinical reality of a patient treatment journey within the entire motor fluctuation spectrum. As this was a pharmacokinetics study in nature with relevant exploratory motor complication outcomes, there was a need to maintain some key fixed variables to minimize bias when analyzing the results; therefore, the same adjustments on LD intakes and dosing intervals were applied to all patients. It should also be noted that the impact of a higher LD bioavailability on patients' activities of daily living and quality of life was not evaluated. The current study did not evaluate the effect of opicapone on extended‐release LD; however, this should be assessed in future studies.

In summary, this study presents evidence from a pharmacokinetic and efficacy standpoint that the modifications in LD plasma profile following the addition of opicapone 50 mg to LD/CD are associated with a reduction in motor fluctuations at a lower daily LD dose (four or five intakes) when compared with a LD/CD‐only regimen with a higher total daily LD dose. These findings may have implications for the treatment of patients with early signs of wearing‐off where adding opicapone 50 mg may be considered as an alternative option to increasing the LD dosing frequency and total daily dose.^29^ Our findings also suggest that a higher number of intakes of LD may influence the response to this type of adjuvant treatment. This will allow physicians to individually adjust the LD regimen that best suits patients' needs without necessarily having to start increasing the LD dose. Such a treatment regimen may not only be useful as a first approach to treat motor fluctuations when diagnosed but also may enable physicians to optimize the management of motor fluctuations at a later stage.

## Authorship

All named authors meet the International Committee of Medical Journal Editors criteria for authorship for this article, take responsibility for the integrity of the work as a whole, and have given their approval for this version to be published.

## Author Roles

(1) Research Project: A. Conception, B. Organization, C. Execution; (2) Manuscript Preparation: A. Writing of the First Draft, B. Review and Critique.

J.J.F.: 1A, 1B, 2A, 2B

W.P.: 1A, 1B, 2B

O.R.: 1A, 1B, 2B

F.S.: 1A, 1B, 2B

A.A.: 1A, 1B, 2B

J.M.: 1A, 1B, 2B

B.G.: 1A, 1B, 2B

J.‐F.R.: 1A, 1B, 2B

P.S.S.: 1A, 1B, 2B

## Disclosures

J.J.F. has provided consultancy for Ipsen, GlaxoSmithKline, Novartis, Teva, Lundbeck, Solvay, Abbott, BIAL, Merck‐Serono, and Merz; and has received grants from GlaxoSmithKline, Grunenthal, Teva, and Fundação Merck Sharp Dohme (MSD). W.P. has received lecture fees and honoraria for consultancy in relation to clinical drug development programs from Alterity, AbbVie, Affiris, AstraZeneca, Axovant, BIAL, Biogen, Britannia, Lilly, Lundbeck, NeuroDerm, Neurocrine, Denali Pharmaceuticals, Orion Pharma, Roche, Stada, Sunovion, Takeda, UCB, and Zambon as well as grant support from The Michael J. Fox Foundation and the EU Seventh Framework Programme (FP7) and Horizon 2020 programs. O.R. has participated in advisory boards and/or provided consultancy for AbbVie, Adamas, Acorda, Addex, AlzProtect, ApoPharma, AstraZeneca, Axovant, BIAL, Biogen, Britannia, Buckwang, CereSpir, Clevexel, Denali, INC Research, IPMDS, Lundbeck, Lupin, Merck, MundiPharma, NeurATRIS, NeuroDerm, Novartis, ONO Pharma, Osmotica, Parexel, Pfizer, Prexton Therapeutics, Quintiles, Roche, Sanofi, Servier, Sunovion, Theranexus, Takeda, Teva, UCB, Vectura, Watermark Research, XenoPort, XO, and Zambon; received grants from Agence Nationale de la Recherche, CHU (Centre Hospitalier Universitaire) de Toulouse, France‐Parkinson, INSERM (Institut National de la Santé Et de la Recherche Médicale) Recherche Clinique Translationnelle, The Michael J. Fox Foundation, Programme Hospitalier de Recherche Clinique, European Commission (FP7, H2020), Cure Parkinson UK; and received a grant to participate in a symposium and contribute to the review of an article by the International Parkinson and Movement Disorder Society. F.S. has received compensation for consultancy and speaker‐related activities from Lundbeck, UCB, Chiesi, Zambon, Britannia, Cynapsus, Sunovion, Kyowa, Abbvie, Neuroderm, Biogen, and BIAL. A.A. has received compensation for consultancy and speaker‐related activities from UCB, Boehringer Ingelheim, Britannia, AbbVie, Zambon, BIAL, NeuroDerm, Theravance Biopharma, and Roche and he receives research support from Chiesi Pharmaceuticals, Lundbeck, Horizon 2020–Grant 825,785, Horizon2020 Grant 101,016,902, Ministry of Education University and Research Grant ARS01_01081, and the Cariparo Foundation. He serves as consultant for Boehringer Ingelheim for legal cases on pathological gambling, owns Patent WO2015110261‐A1, and owns shares in PD Neurotechnology Limited. J.M., B.G., J.‐F.R., and P.S.S. are/were employees of BIAL–Portela & Cª, S.A.

## Supporting information


**Table S1** The 12‐hour *on*‐time/*off*‐time data reported on pharmacokinetics days following 2‐week, five‐intake (every 3 hours apart) daily oral administrations of levodopa/carbidopa 500/125 mg without opicapone compared with 2‐week, four‐intake (every 4 hours) daily oral administrations of levodopa/carbidopa 400/100 mg plus once‐daily opicapone 50 mg or compared with 2‐week, five‐intake (every 3 hours) daily oral administrations of levodopa/carbidopa 400/100 mg plus once‐daily opicapone 50 mg.
**Table S2.** The 24‐hour Hauser *on*‐time/*off*‐time diary data following 2‐week, five‐intake (every 3 hours apart) daily oral administrations of levodopa/carbidopa 500/125 mg compared with 2‐week, four‐intake (every 4 hours) daily oral administrations of levodopa/carbidopa 400/100 mg plus once‐daily 50 mg opicapone or 2‐week, five‐intake (every 3 hours) daily oral administrations of levodopa/carbidopa 400/100 mg plus once‐daily opicapone 50 mg.
**Table S3.** The 3‐O‐methyldopa pharmacokinetic parameters following 2‐week, five‐intake (every 3 hours) daily oral administrations of levodopa/carbidopa 500/125 mg without opicapone compared with 2‐week, four‐intake (every 4 hours) daily oral administrations of levodopa/carbidopa 400/100 mg plus once‐daily opicapone 50 mg or compared with 2‐week, five‐intake (every 3 hours) daily oral administrations of levodopa/carbidopa 400/100 mg plus once‐daily opicapone 50 mg.
**Table S4.** Paired *t* test analysis for main 3‐O‐methyldopa pharmacokinetics parameters following 2‐week, five‐intake (every 3 hours) daily oral administrations of levodopa/carbidopa 500/125 mg without opicapone compared with 2‐week, four‐intake (every 4 hours) daily oral administrations of levodopa/carbidopa 400/100 mg plus once‐daily opicapone 50 mg or compared with 2‐week, five‐intake (every 3 hours) daily oral administrations of levodopa/carbidopa 400/100 mg plus once‐daily opicapone 50 mg.
**Table S5.** Summary of treatment‐emergent adverse events.
**Figure S1.** The 12‐hour *on*‐time/*off*‐time data reported on pharmacokinetics days superimposed to the mean levodopa plasma profile versus time following: 2‐week, five‐intake (every 3 hours) daily oral administrations of levodopa/carbidopa 500/125 mg without opicapone (A) compared with 2‐week, four‐intake (every 4 hours) daily oral administrations of levodopa/carbidopa 400/100 mg plus once‐daily opicapone 50 mg (B); 2‐week, five‐intake (every 3 hours) daily oral administrations of levodopa/carbidopa 500/125 mg without opicapone (C) compared with 2‐week, five‐intake (every 3 hours) daily oral administrations of levodopa/carbidopa 400/100 mg plus once‐daily opicapone 50 mg (D). LD/CD, levodopa/carbidopa; OPC, opicapone; black arrows, time to *on*; orange bars, *on*‐state periods; blue line, time of best *on*.
**Figure S2.** Mean 3‐O‐methyldopa plasma profile versus time following 2‐week, five‐intake (every 3 hours) daily oral administrations of LD/CD 500/125 mg compared with 2‐week, four‐intake (every 4 hours) daily oral administrations of LD/CD 400/100 mg plus once‐daily opicapone 50 mg (A) or compared with 2‐week, five‐intake (every 3 hours) daily oral administrations of LD/CD 400/100 mg plus once‐daily opicapone 50 mg (B). LD/CD, levodopa/carbidopa; OPC, opicapone.
**Figure S3.** PGI‐C following 2‐week, four‐intake (every 4 hours) daily oral administrations of LD/CD 400/100 mg plus once‐daily opicapone 50 mg (A) and following 2‐week, five‐intake (every 3 hours) daily oral administrations of LD/CD 400/100 mg plus once‐daily opicapone 50 mg (B). LD/CD, levodopa/carbidopa; OPC, opicapone; PGI‐C, Patient Global Impression of Change.
**Figure S4:** Response to levodopa therapy in relation to levodopa pharmacokinetics. AUC, area under the curve, C_max_, maximum observed plasma concentration; C_min_, minimum observed plasma concentration.

## Data Availability

The data that support the findings of this study are available from the corresponding author upon reasonable request.
