# Supplementary material for: Effect of Opicapone on Levodopa Pharmacokinetics in Patients with Fluctuating Parkinson's Disease
Source: Mov Disord. 2022 Aug 31;37(11):2272–83. doi: 10.1002/mds.29193 (PMC9804871; doi:10.1002/mds.29193)
Supplement: Supplementary file 1 — Table S1 The 12‐hour on‐time/off‐time data reported on pharmacokinetics days following 2‐week, five‐intake (every 3 hours apart) daily oral administrations of levodopa/carbidopa 500/125 mg without opicapone compared with 2‐week, four‐intake (every 4 hours) daily oral administrations of levodopa/carbidopa 400/100 mg plus once‐daily opicapone 50 mg or compared with 2‐week, five‐intake (every 3 hours) daily oral administrations of levodopa/carbidopa 400/100 mg plus once‐daily opicapone 50 mg. Table S2. The 24‐hour Hauser on‐time/off‐time diary data following 2‐week, five‐intake (every 3 hours apart) daily oral administrations of levodopa/carbidopa 500/125 mg compared with 2‐week, four‐intake (every 4 hours) daily oral administrations of levodopa/carbidopa 400/100 mg plus once‐daily 50 mg opicapone or 2‐week, five‐intake (every 3 hours) daily oral administrations of levodopa/carbidopa 400/100 mg plus once‐daily opicapone 50 mg. Table S3. The 3‐O‐methyldopa pharmacokinetic parameters following 2‐week, five‐intake (every 3 hours) daily oral administrations of levodopa/carbidopa 500/125 mg without opicapone compared with 2‐week, four‐intake (every 4 hours) daily oral administrations of levodopa/carbidopa 400/100 mg plus once‐daily opicapone 50 mg or compared with 2‐week, five‐intake (every 3 hours) daily oral administrations of levodopa/carbidopa 400/100 mg plus once‐daily opicapone 50 mg. Table S4. Paired t test analysis for main 3‐O‐methyldopa pharmacokinetics parameters following 2‐week, five‐intake (every 3 hours) daily oral administrations of levodopa/carbidopa 500/125 mg without opicapone compared with 2‐week, four‐intake (every 4 hours) daily oral administrations of levodopa/carbidopa 400/100 mg plus once‐daily opicapone 50 mg or compared with 2‐week, five‐intake (every 3 hours) daily oral administrations of levodopa/carbidopa 400/100 mg plus once‐daily opicapone 50 mg. Table S5. Summary of treatment‐emergent adverse events. Figure S1. The 12‐hour on‐time/off‐time d [file MDS-37-2272-s001.docx]

**Table S1. 12-hour ON-/OFF-time data reported on pharmacokinetics days following a 2-week 5-intake (every 3 hours apart) daily oral administrations of levodopa/carbidopa 500/125mg without opicapone compared with a 2-week 4-intake (every 4 hours) daily oral administrations of levodopa/carbidopa 400/100mg plus once-daily opicapone 50mg or compared with a 2-week 5-intake (every 3 hours) daily oral administrations of levodopa/carbidopa 400/100mg plus once-daily opicapone 50mg**

|  | **Time to ON (min)**  **n=12** | **12-hour Time to ON (min)**  **n=12** | **Time to Best-ON (min)**  **n=12** | **12-hour Time to Best-ON (min)**  **n=12** | **OFF-time (min)**  **n=12** | **12-hour OFF-time (min)**  **n=12** | **ON-time (min)**  **n=12** | **12-hour ON-time (min)**  **n=12** | **ON-time Without Dyskinesia**  **(min)**  **n=12** | **12-hour ON-time Without Dyskinesia**  **(min)**  **n=12** | **ON-time**  **With Dyskinesia (min)**  **n=12** | **12-hour ON-time With Dyskinesia (min)**  **n=12** |
| --- | --- | --- | --- | --- | --- | --- | --- | --- | --- | --- | --- | --- |
| **5-intake LD/CD 500/125mg vs 4-intake LD/CD 400/100mg plus OPC 50mg** | | | | | | | | | | | | |
| **5-intake LD/CD 500/125mg** | | | | | | | | | | | | |
| 1^st^ LD/CD intake | 38.9 | 46.14  (12.38) | 55.0 | 59.22  (14.92) | 79.8 | 353.3  (80.46) | 100.3 | 347.5  (92.53) | 92.8 | 322.5  (106.27) | 45.0 | 60.0  (45.14) |
|  | (17.98) |  | (26.80) |  | (30.91) |  | (30.91) |  | (35.71) |  | (14.14) |  |
| 2^nd^ LD/CD intake | 50.0 |  | 58.8 |  | 106.9 |  | 73.1 |  | 67.3 |  | 23.3 |  |
|  | (34.84) |  | (31.42) |  | (23.85) |  | (23.85) |  | (26.93) |  | (5.77) |  |
| 3^rd^ LD/CD intake | 40.5 |  | 62.0 |  | 89.5 |  | 90.5 |  | 81.8 |  | 23.8 |  |
|  | (19.81) |  | (24.29) |  | (34.67) |  | (34.67) |  | (38.88) |  | (4.79) |  |
| 4^th^ LD/CD intake | 42.1 |  | 57.9 |  | 84.6 |  | 91.3 |  | 87.5 |  | 22.5 |  |
|  | (17.64) |  | (19.82) |  | (29.50) |  | (31.20) |  | (33.34) |  | (10.61) |  |
| **4-intake LD/CD 400/100mg plus OPC 50mg** | | | | | | | | | | | | |
| 1^st^ LD/CD intake | 36.8 | 44.58  (24.47) | 50.4 | 51.25  (22.80) | 121.4 | 302.1  (106.31) | 118.6 | 374.6  (112.7) | 115.0 | 357.1  (110.30) | 20.0 | 52.5  (46.28) |
|  | (12.70) |  | (25.36) |  | (41.90) |  | (41.90) |  | (41.83) |  | (0.00) |  |
| 2^nd^ LD/CD intake | 41.8 |  | 47.9 |  | 108.2 |  | 131.8 |  | 119.5 |  | 45.0 |  |
|  | (30.84) |  | (28.88) |  | (35.73) |  | (35.73) |  | (39.65) |  | (27.84) |  |
| 3^rd^ LD/CD intake | 45.0 |  | 55.4 |  | 91.7 |  | 145.0 |  | 142.1 |  | 17.5 |  |
|  | (25.50) |  | (26.41) |  | (31.86) |  | (30.60) |  | (29.42) |  | (3.54) |  |
| **5-intake LD/CD 500/125mg vs 5-intake LD/CD 400/100mg plus OPC 50mg** | | | | | | | | | | | | |
| **5-intake LD/CD 500/125mg** | | | | | | | | | | | | |
| 1^st^ LD/CD intake | 41.5 | 43.13  (11.32) | 62.7 | 55.46  (14.86) | 88.5 | 348.1  (103.9) | 91.5 | 320.1  (83.80) | 79.2 | 280.5  (71.44) | 33.8 | 72.5  (24.24) |
|  | (9.91) |  | (29.28) |  | (26.54) |  | (26.54) |  | (27.39) |  | (17.97) |  |
| 2^nd^ LD/CD intake | 36.0 |  | 51.7 |  | 103.0 |  | 77.0 |  | 71.5 |  | 27.5 |  |
|  | (16.30) |  | (26.31) |  | (22.88) |  | (22.88) |  | (22.98) |  | (3.54) |  |
| 3^rd^ LD/CD intake | 44.5 |  | 50.0 |  | 86.5 |  | 93.5 |  | 74.5 |  | 31.7 |  |
|  | (22.66) |  | (17.06) |  | (19.73) |  | (19.73) |  | (23.62) |  | (6.83) |  |
| 4^th^ LD/CD intake | 52.5 |  | 57.5 |  | 96.0 |  | 81.0 |  | 75.5 |  | 27.5 |  |
|  | (15.68) |  | (21.05) |  | (34.22) |  | (33.48) |  | (31.31) |  | (3.54) |  |
| **5-intake LD/CD 400/100mg plus OPC 50mg** | | | | | | | | | | | | |
| 1^st^ LD/CD intake | 39.5 | 33.83  (20.84) | 53.3 | 49.37  (19.14) | 64.0 | 239.5  (92.27) | 116.0 | 460.5  (135.04) | 110.5 | 412.5  (147.63) | 27.5 | 68.6  (33.26) |
|  | (18.33) |  | (22.29) |  | (27.47) |  | (27.47) |  | (37.89) |  | (24.75) |  |
| 2^nd^ LD/CD intake | 28.0 |  | 48.2 |  | 62.0 |  | 118.0 |  | 101.5 |  | 41.3 |  |
|  | (19.03) |  | (27.50) |  | (41.04) |  | (41.04) |  | (50.11) |  | (40.08) |  |
| 3^rd^ LD/CD intake | 29.4 |  | 48.5 |  | 57.8 |  | 122.2 |  | 109.4 |  | 28.8 |  |
|  | (19.76) |  | (20.28) |  | (31.73) |  | (31.73) |  | (41.34) |  | (7.50) |  |
| 4^th^ LD/CD intake | 35.0 |  | 52.5 |  | 61.5 |  | 116.5 |  | 102.0 |  | 29.0 |  |
|  | (34.16) |  | (29.46) |  | (37.64) |  | (39.65) |  | (44.73) |  | (9.62) |  |

LD/CD, levodopa/carbidopa; OPC, opicapone; All values are expressed as mean (SD); SD, standard deviation

**Table S2. 24-hour Hauser ON-/OFF-time diary data following a 2-week 5-intake (every 3 hours apart) daily oral administrations of levodopa/carbidopa 500/125mg compared with a 2-week 4-intake (every 4 hours) daily oral administrations of levodopa/carbidopa 400/100mg plus once-daily 50mg opicapone or a 2-week 5-intake (every 3 hours) daily oral administrations of levodopa/carbidopa 400/100mg plus once-daily opicapone 50mg**

|  | **Total OFF-time (min)**  **n=12** | **Total ON-time (min)**  **n=12** | **ON-time Without Dyskinesia**  **(min)**  **n=12** | **ON-time With Non-Troublesome Dyskinesia (min)**  **n=12** | **ON-time With Troublesome Dyskinesia (min)**  **n=12** |
| --- | --- | --- | --- | --- | --- |
|  | **5-intake LD/CD 500/125mg vs 4-intake LD/CD 400/100mg plus OPC 50mg** | | | |  |
| **5-intake LD/CD 500/125mg** | 442.50 (96.3) | 488.3 (110.9) | 411.7 (122.6) | 86.7 (72.6) | 35.0 (26.5) |
|  |  |  |  |  |  |
|  |  |  |  |  |  |
| **4-intake LD/CD 400/100mg plus OPC 50mg** | 400.0 (116.4) | 540.8 (121.4) | 471.7 (153.0) | 81.1 (78.7) | 25.0 (20.8) |
|  |  |  |  |  |  |
|  | **5-intake LD/CD 500/125mg vs 5-intake LD/CD 400/100mg plus OPC 50mg** | | | |  |
| **5-intake LD/CD 500/125mg** | 431.70 (100.3) | 519.2 (91.2) | 446.7 (110.0) | 66.4 (48.0) | 20.0 (24.49) |
|  |  |  |  |  |  |
|  |  |  |  |  |  |
| **5-intake LD/CD 400/100mg plus OPC 50mg** | 338.3 (111.3) | 622.5 (106.7) | 547.5 (147.6) | 73.6 (62.3) | 12.9 (13.8) |

LD/CD, levodopa/carbidopa; OPC, opicapone; All values are expressed as mean (SD); SD, standard deviation

**Table S3. 3-O-methyldopa pharmacokinetic parameters following 2-week 5-intake (every 3 hours) daily oral administrations of levodopa/carbidopa 500/125mg without opicapone compared with 2-week 4-intake (every 4 hours) daily oral administrations of LD/CD 400/100mg plus once-daily opicapone 50mg or compared with 2-week 5-intake (every 3 hours) daily oral administrations of levodopa/carbidopa 400/100mg plus once-daily opicapone 50mg**

|  | **C_max_ (ng/mL)**  **n=12** | **C_max,max_ (ng/mL)**  **n=12** | **t_max_**  **(h)**  **n=12** | **C_min_**  **(ng/mL)**  **n=12** | **AUCτ (h*ng/mL)**  **n=12** | **AUC_total_**  **(h*ng/mL)**  **n=12** | **t_1/2_**  **(h)**  **n=12** | **FI**  **(%)**  **n=12** |
| --- | --- | --- | --- | --- | --- | --- | --- | --- |
| **5-intake LD/CD 500/125mg vs 4-intake LD/CD 400/100mg plus OPC 50mg** | | | | | | | | |
| **5-intake LD/CD 500/125mg** | | | | | | | | |
| 1^st^ LD/CD intake | 3453 | 4447 (29.2) | 1.75 | 3021 | 10068^a^ | 43080 (31.8) | 13.5^d^ | 12.9^a^ |
|  | (34.0) |  | (0–2.5) | (35.3) | (39.4) |  | (63.8) | (36.9) |
| 2^nd^ LD/CD intake | 3619 |  | 2.5 | 3077 | - |  | - | - |
|  | (33.9) |  | (2.0–2.92) | (35.5) | - |  | - | - |
| 3^rd^ LD/CD intake | 4011 |  | 2.71 | 3437 | 14278^b^ |  | - | 9.87^b^ |
|  | (28.3) |  | (0.5–2.92) | (32.3) | (24.0) |  | - | (92.5) |
| 4^th^ LD/CD intake | 4435 |  | 2.5 | 3766 | 13519^c^ |  | - | 14.6^c^ |
|  | (29.5) |  | (0.5–2.92) | (29.3) | - |  | - | - |
| **4-intake LD/CD 400/100mg plus OPC 50mg** | | | | | | | | |
| 1^st^ LD/CD intake | **521** | 595 (35.8) | 1.5 | 457 | 1840^e^ | 6065 (35.2) | 14.2^d^ | 14.2^e^ |
|  | **(36.9)** |  | (0–2.5) | (37.1) | (36.3) |  | (16.9) | (118.5) |
| 2^nd^ LD/CD intake | **530** |  | 3.46 | 436 | 2242^f^ |  | 39.0^c^ | 36.2^f^ |
|  | **(33.7)** |  | (1.5–3.92) | (36.5) | (23.0) |  | - | (74.9) |
| 3^rd^ LD/CD intake | **581** |  | 3.92 | 485 | - |  | - | - |
|  | **(36.0)** |  | (2.5–3.97) | (34.2) | - |  | - | - |
|  |  | **5-intake LD/CD 500/125mg vs 5-intake LD/CD 400/100mg plus OPC 50mg** | | | | |  |  |
| **5-intake LD/CD 500/125mg** | | | | | | | | |
| 1^st^ LD/CD intake | 3191 | 4300 (22.4) | 1.5 | 2868 | 8991^g^ | 40656 (24.2) | 15.2^b^ | 8.63^g^ |
|  | (25.5) |  | (1.0–2.92) | (24.3) | (33.0) |  | (51.2) | (22.9) |
| 2^nd^ LD/CD intake | 3406 |  | 2.5 | 2907 | 9002^d^ |  | 9.43^c^ | 19.7^d^ |
|  | (22.6) |  | (0.5–2.95) | (25.9) | (13.7) |  | - | (47.4) |
| 3^rd^ LD/CD intake | 3875 |  | 2.5 | 3151 | 11951^c^ |  | - | 10.3^c^ |
|  | (21.9) |  | (1.0–2.92) | (27.3) | - |  | - | - |
| 4^th^ LD/CD intake | 4170 |  | 2.71 | 3586 | - |  | - | - |
|  | (25.6) |  | (2.0–2.97) | (26.9) | - |  | **-** | **-** |
| **5-intake LD/CD 400/100mg plus OPC 50mg** | | | | | | | | |
| 1^st^ LD/CD intake | 484 |  | 1.0 | 451 | 1180^h^ | 5818 (39.9) | 12.8^a^ | 8.76^h^ |
|  | (39.9) | 575 (36.5) | (0–2.92) | (42.5) | (28.5) |  | (27.7) | (33.8) |
| 2^nd^ LD/CD intake | 485 |  | 2.0 | 446 | - |  | - | - |
|  | (39.8) |  | (1.5–2.98) | (40.5) | - |  | - | - |
| 3^rd^ LD/CD intake | 514 |  | 2.25 | 470 | 1568^c^ |  | - | 4.02^c^ |
|  | (211) |  | (1.0–2.92) | (41.1) | - |  | - | - |
| 4^th^ LD/CD intake | 575 |  | 2.92 | 498 | - |  | - | - |
|  | (36.6) |  | (2.0–2.95) | (40.3) | - |  | - | - |

LD/CD, levodopa/carbidopa; OPC, opicapone; All values are expressed as mean (%CV) except for t_max_ values that are expressed as median (range); ^a^5 patients; ^b^3 patients; ^c^1 patient; ^d^4 patients; ^e^11 patients; ^f^2 patients; ^g^7 patients; ^h^6 patients. AUCτ, area under the concentration-time curve (AUC) over the dosing interval; AUC_total_, AUC from zero hours to the last measurable time point; C_max_, maximum observed plasma concentration; C_max,max_, maximum C_max_ observed; C_min_, minimum observed plasma concentration; C_min,min_, minimum C_min_ observed (excluding first pre-dose); FI, fluctuation index (calculated as: [(C_max_ – C_min_)/C_avg_]*100); CV, coefficient of variation; t_max_, time taken to reach C_max_ ; SD, standard deviation t_1/2_, terminal plasma half-life

**Table S4. Paired t-test analysis for main 3-O-methyldopa pharmacokinetics parameters following a 2-week 5-intake (every 3 hours) daily oral administrations of levodopa/carbidopa 500/125mg without opicapone compared with a 2-week 4-intake (every 4 hours) daily oral administrations of levodopa/carbidopa 400/100mg plus once-daily opicapone 50mg or compared with a 2-week 5-intake (every 3 hours) daily oral administrations of levodopa/carbidopa 400/100mg plus once-daily opicapone 50mg**

| **Parameter** | | **N** | **Geometric Mean (90% CI)** | | | **Geometric Mean Ratio (GMR)** | | | |
| --- | --- | --- | --- | --- | --- | --- | --- | --- | --- |
|  |  |  | **Reference**  **(5-intake LD/CD 500/125mg)** | | **Test**  **(4/5-intake LD/CD 400/100mg**  **+OPC 50mg)** | **Estimate** | **90% CI** | | ***p value*** |
| **5-intake levodopa/carbidopa 500/125mg without opicapone vs 4-intake levodopa/carbidopa 400/100mg plus opicapone 50mg** | | | | | | | | | |
| AUC_total_ (h•ng/mL) | | 12 | 41051 (34603, 48700) | | 5759 (4851, 6837) | 0.14 | 0.13, 0.15 | | *<0.0001* |
| C_max,max_ (ng/mL) | | 12 | 4264 (3631, 5006) | | 563 (471, 672) | 0.13 | 0.12, 0.14 | | *<0.0001* |
| **5-intake levodopa/carbidopa 500/125mg without opicapone vs 5-intake LD/CD 400/100mg plus opicapone 50mg** | | | | | | | | | |
| AUC_total_ (h•ng/mL) | 12 | | 39650 (35181, 44687) | 5450 (4499, 6601) | | 0.14 | 0.12, 0.16 | *<0.0001* | |
| C_max,max_ (ng/mL) | 12 | | 4201 (3733, 4727) | 541 (448, 654) | | 0.13 | 0.11, 0.15 | *<0.0001* | |

LD/CD, levodopa/carbidopa; C_max_, maximum observed plasma concentration; C_max,max_, maximum C_max_ observed; AUC_total_, area under the curve (AUC) from zero hours to the last measurable time point

**Table S5. Summary of TEAEs**

|  | 5-intake levodopa/carbidopa 500/125mg vs 4-intake levodopa/carbidopa 400/100mg plus opicapone 50mg  **n=12** | 5-intake levodopa/carbidopa 500/125mg vs 5-intake levodopa/carbidopa 400/100mg plus opicapone 50mg  **n=12** |
| --- | --- | --- |
| Number of TEAEs, n (%) | 2 (16.7) | 0 |
| Patients with at least one TEAEs, n (%) | 2 (16.7) | 0 |
| Number of serious TEAEs, n (%) | 0 | 0 |
| Patients with at least one serious TEAEs, n (%) | 0 | 0 |
| Number of TEAEs leading to discontinuation, n (%) | 0 | 0 |

TEAE, treatment-emergent adverse event

| **A**  **** | **B**  **** |
| --- | --- |
| **C**  **** | **D**  **** |

**Figure S1. 12-hour ON-/OFF-time data reported on pharmacokinetics days superimposed to the mean levodopa plasma profile versus time following: 2-week 5-intake (every 3 hours) daily oral administrations of levodopa/carbidopa 500/125mg without opicapone (A) compared with 2-week 4-intake (every 4 hours) daily oral administrations of levodopa/carbidopa 400/100mg plus once-daily opicapone 50mg (B); 2-week 5-intake (every 3 hours) daily oral administrations of levodopa/carbidopa 500/125mg without opicapone (C) compared with 2-week 5-intake (every 3 hours) daily oral administrations of levodopa/carbidopa 400/100mg plus once-daily opicapone 50mg (D).** LD/CD, levodopa/carbidopa; OPC, opicapone; black arrows, time to ON; orange bars, ON state periods; blue line, time of Best ON

| **A**  **** | **B**  **** |
| --- | --- |

**Figure S2. Mean 3-O-methyldopa plasma profile versus time following a 2-week 5-intake (every 3 hours) daily oral administrations of LD/CD 500/125mg compared with a 2-week 4-intake (every 4 hours) daily oral administrations of LD/CD 400/100mg plus once-daily opicapone 50mg (A) or compared with a 2-week 5-intake (every 3 hours) daily oral administrations of LD/CD 400/100mg plus once-daily opicapone 50mg (B).** LD/CD, levodopa/carbidopa; OPC, opicapone

**A 4-intake LD/CD 400/100mg plus once-daily opicapone 50mg**

**B 5-intake LD/CD 400/100mg plus once-daily opicapone 50mg**

**Figure S3**. **Patient Global Impression of Change following a 2-week 4-intake (every 4 hours) daily oral administrations of LD/CD 400/100mg plus once-daily opicapone 50mg (A); and following a 2-week 5-intake (every 3 hours) daily oral administrations of LD/CD 400/100mg plus once-daily opicapone 50mg (B).**  LD/CD, levodopa/carbidopa; OPC, opicapone; OPC, opicapone, PGI-C, Patient Global Impression of Change

**
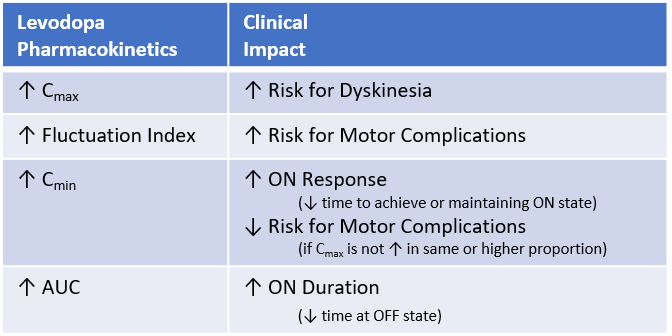
**

**Figure S4: Response to levodopa therapy in relation to levodopa pharmacokinetics.** AUC, area under the curve, C_max_, maximum observed plasma concentration; C_min_, minimum observed plasma concentration
